# Gender disparities in lung cancer and social determinants of environmental risk: a geospatial analysis across Illinois counties

**DOI:** 10.3389/fpubh.2025.1676853

**Published:** 2026-01-23

**Authors:** Cheng-chia Brian Chen, Angela M. McComas, Echu Liu, Jorge Villegas

**Affiliations:** 1 College of Health, Science and Technology, University of Illinois Springfield, Springfield, IL, United States; 2 Department of Pharmacology, Southern Illinois University School of Medicine, Springfield, IL, United States; 3 Department of Health Management and Policy, Saint Louis University, Saint Louis, MO, United States; 4 College of Business and Management, University of Illinois Springfield, Springfield, IL, United States

**Keywords:** gender disparities, geospatial analysis, housing vulnerability, lung cancer, radon exposure, smoking prevalence, social determinants of health

## Abstract

**Background:**

Lung cancer remains the leading cause of cancer-related death in Illinois. While tobacco use is the primary risk factor, environmental exposures and social determinants of health (SDOH)—such as housing conditions and socioeconomic vulnerability—also shape cancer risk across geographic and demographic groups.

**Objectives:**

This study examines the impact of radon exposure, smoking prevalence, and housing-related vulnerability on lung cancer incidence among males and females across all 102 counties in Illinois.

**Methods:**

We conducted comprehensive county-level analyses of social determinants of health indicators to examine their influence on lung cancer using sex-stratified, age-adjusted lung cancer incidence rates (2017–2021 average). Additionally, further examinations using spatial analyses with bi-variate choropleth mapping approaches were performed to target underserved populations. Independent variables included radon exposures, such as average residential radon levels, adult smoking prevalence, and housing-related indicators from the CDC/ATSDR Social Vulnerability Index (SVI). Multivariate linear regression models were developed separately for males and females. Geospatial bivariate choropleth maps were created to highlight overlapping hotspots of cancer burden and environmental exposures.

**Results:**

Smoking prevalence was the strongest predictor of lung cancer for both sexes. Radon exposure significantly predicted female lung cancer incidence ( β = 0.195, *p =* 0.027), whereas housing-related vulnerability was significant only for males ( β = 0.234, *p* = 0.009). Geospatial analyses identified priority regions for targeted interventions, including high-risk radon zones in northwestern Illinois for females and housing-vulnerable counties in central and eastern Illinois for males.

**Conclusion:**

This study highlights the role of sex-specific interactions between environmental risk and social vulnerability in contributing to lung cancer disparities. Integrating geospatial and ecological analyses through the SDOH lens can inform tailored public health interventions that advance health equity and environmental justice in cancer prevention.

## 1 Introduction

Lung cancer remains the leading cause of cancer-related mortality in Illinois, with a significant incidence difference between sexes (Male 70.6 per 100,000, Female 54.3 per 100,000) and between non-Hispanic black males (91.3 per 100,000) and other ethnic and racial groups. Patterns of mortality rate in the state mirror incidence as black males are more likely to die of the disease (63.2 per 100,000) than all males (46.9 per 100,000) or females (33.2 per 100,000). Geographically, data suggest high levels of inequity, as the incidence of lung cancer in certain counties (i.e., Hardin 133.6, Pulaski 112.1, Brown 108.9, and Franklin 108.3 per 100,000) is significantly higher than the state’s average (63.7 per 100,000) (1). These data suggest that in Illinois, cancer incidence and mortality are linked to disparities rooted in environmental exposures and social determinants of health (SDOH), as researchers have found strong links and interactions between cancer incidence and mortality and race (2), SDOHs (3), and geographical features such as rurality and persistent poverty (4, 5).

While tobacco smoking accounts for approximately 80% of lung cancer deaths in the United States, indoor radon exposure is the second leading cause, responsible for an estimated 21,000 deaths annually (6, 7). The risk of radon-induced lung cancer is influenced by both the concentration of radon progeny in indoor air and the duration of exposure (8, 9). Prolonged indoor occupancy in homes with elevated radon levels significantly increases individual risk (9, 10). According to the EPA map designation (Supplementary File S1), large portions of Illinois fall within Zone 1 (highest potential) and Zone 2 (moderate potential) radon exposure areas, indicating that residents face elevated indoor radon levels that significantly increase their lifetime risk of developing lung cancer. In Illinois, 45% of homes test at or above the EPA radon action level (11). However, testing and mitigation rates vary substantially by geography and socioeconomic status, with rural and low-income communities often having the lowest testing rates (12).

Broader structural conditions shape these environmental risks. SDOH—including housing quality, economic stability, and geographic location—play a critical role in influencing exposure to environmental carcinogens. Poor housing conditions and limited access to radon testing or remediation services can increase vulnerability to indoor pollutants. The Centers for Disease Control and Prevention (CDC) Social Vulnerability Index (SVI) aggregates socioeconomic and housing-related indicators to assess the risk to the population that lives in specific census tracts by using the index variables related to socioeconomic status, household characteristics, racial and ethnic minority status, and housing type and transportation (13). In particular, the last dimension is relevant to understanding geography-specific risks, such as radon, as it uses variables such as mobile homes and other social vulnerability-housing type factors associated with lung cancer (14). However, few studies have examined how environmental exposures, such as radon, interact with social vulnerability, particularly when stratified by sex (13, 15).

Emerging evidence suggests that men and women experience distinct patterns of environmental risk, which contribute to sex-based disparities in lung cancer. Females may be more biologically susceptible to tobacco smoke carcinogens and radon exposure, and paradoxically, they exhibit higher lung cancer incidence even among nonsmokers, suggesting additional environmental or behavioral risk pathways (16). In contrast, males are more likely to encounter occupational and housing-related exposures linked to social vulnerability, particularly in underserved environments (17, 18).

The interplay between environmental exposures and socioeconomic conditions contributes significantly to sex-specific health disparities (19, 20). Housing instability and lower socioeconomic status, as measured by the SVI, can increase exposure to indoor carcinogens, such as radon, particularly in communities with limited access to remediation (21, 22). Gender-specific behaviors and occupational roles further shape these risks (21). This study builds on prior environmental health research by examining how these intersecting factors—across physical, social, and behavioral domains—shape geographic and sex-based variation in lung cancer outcomes (23, 24).

Following deeper reviews of the literature (25–29), we hypothesized that radon exposure and smoking prevalence would be significantly more predictive of lung cancer incidence among females than among males. Although researchers in global studies found males having a higher burden of cancer due to Radon exposure (30, 31), other studies have found a stronger etiology of radon and lung cancer among females (16). At the same time, housing-related vulnerability would be more strongly associated with lung cancer incidence among males, as seen in studies of lung cancer and mortality, which suggests that men who live in areas with the most vulnerable SVI have a higher mortality rate due to lung cancer (32). We further anticipated that these patterns would vary spatially across Illinois counties, aligning with regional disparities in environmental risk and social vulnerability.

This study aims to assess sex-specific associations between smoking prevalence, radon exposure, housing-related social vulnerability, and lung cancer incidence across all 102 counties in Illinois. While prior research has examined individual risk factors in isolation, few studies have explored how these environmental and social exposures interact, and even fewer have applied a sex-stratified approach at the population level. By integrating geospatial mapping with multivariate regression, this study offers a novel contribution by revealing region-specific and sex-specific disparities in lung cancer risk. These insights are crucial for informing precision public health strategies, guiding targeted interventions, and addressing environmental health disparities (18), particularly through the lens of environmental justice and social determinants of health.

## 2 Data and methods

The research questions posed in the study, extend the current literature (25–29) by analyzing three independent variables in the prediction model based on their established or hypothesized relationships to lung cancer risk (i.e., dependent variable) and relevance to social determinants of health (SDOH) indicators: (a) adult smoking prevalence, (b) residential radon concentration, and (c) housing-related social vulnerability.

We conducted a retrospective ecological study using county-level data from all 102 counties in Illinois to examine environmental and social determinants of geographic variation in lung cancer incidence. The primary outcome variables were sex-specific, age-adjusted lung cancer incidence rates per 100,000 population, averaged over 5 years (2017–2021), and obtained from the National Cancer Institute’s State Cancer Profiles database. Age adjustment was based on the 2020 U. S. standard population to facilitate standardized comparisons across counties. Due to the complexity of our targeted independent variables, Table 1 summarizes key features of all datasets involved in the present study to help readers understand and compare the covariates of the prediction models, such as social determinants of health indicators and lung cancer risk factors.

**Table 1:** Data source and variable definition summary table.

Variable	Description	Years covered	Source
Lung cancer incidence (sex-specific, age-adjusted)	Age-adjusted incidence rates per 100,000 population, averaged over 5 years. Adjustment based on the 2020 U.S. standard population.	2017–2021	National Cancer Institute (State Cancer Profiles)
Smoking prevalence	Self-reported smoking behavior from population-based surveys; county-level estimates.	2022 (BRFSS-derived)	County Health Rankings & Roadmaps / Illinois BRFSS
Radon exposure	Average county-level residential radon concentrations based on household radon tests.	2003–2019	Illinois Emergency Management Agency (IEMA) Radon Program
Social vulnerability index – housing type	Housing type domain of the CDC/ATSDR SVI (higher = more vulnerable), such as % of mobile homes and % group-quarters population (e.g., nursing homes, group homes, military barracks, correctional facilities, and workers’ dormitories)	2022	CDC / Agency for Toxic Substances and Disease Registry (ATSDR)

Smoking prevalence was obtained from the 2022 County Health Rankings and Roadmaps, which compiles self-reported data from the Illinois Behavioral Risk Factor Surveillance System (BRFSS). Residential radon data were collected from the Illinois Emergency Management Agency (IEMA) Radon Program and reflect household radon test results reported from 2003 to 2019. County-level averages were calculated from individual household readings. Housing-related social vulnerability was measured using the housing type domain of the Social Vulnerability Index (SVI), derived from the CDC and the Agency for Toxic Substances and Disease Registry (ATSDR) in 2022, which ranges from a least-vulnerable score of 0 to a most-vulnerable score of 2 (percentile ranks reported using percentages). This domain measures the SVI-housing and transportation domain-related variables, such as percentile ranks of mobile homes and group-quarters population (e.g., nursing homes, group homes, military barracks, correctional facilities, and workers’ dormitories) (14).

Separate multivariate linear regression models were fitted for females and males using IBM SPSS Statistics (v29), and spatial autocorrelation (Moran’s I) analyses were conducted using Python 3. To account for potential spatial dependencies among counties, a spatial weights matrix was constructed using the queen criterion (i.e., queen-based contiguity). Under this definition, two counties were considered neighbors if they shared any common boundary point. This matrix was row-standardized to ensure that the spatial lag term represented the average value of neighboring observations, facilitating the detection of local spatial clusters through Moran’s I statistic. The purpose of the model-building process was to assess the relationships between lung cancer incidence and environmental and behavioral risk factors. The OLS regression model assumptions for males and females were tested and met. For instance, illustrations of residual diagnostics are provided in Supplementary File S2. Unstandardized and standardized regression coefficients ( β & B) were reported to facilitate comparison across predictors. Additionally, R^2^/AIC/BIC/R^2^ adjusted and VIF values are included in the model result tables.

**Figure 1:**
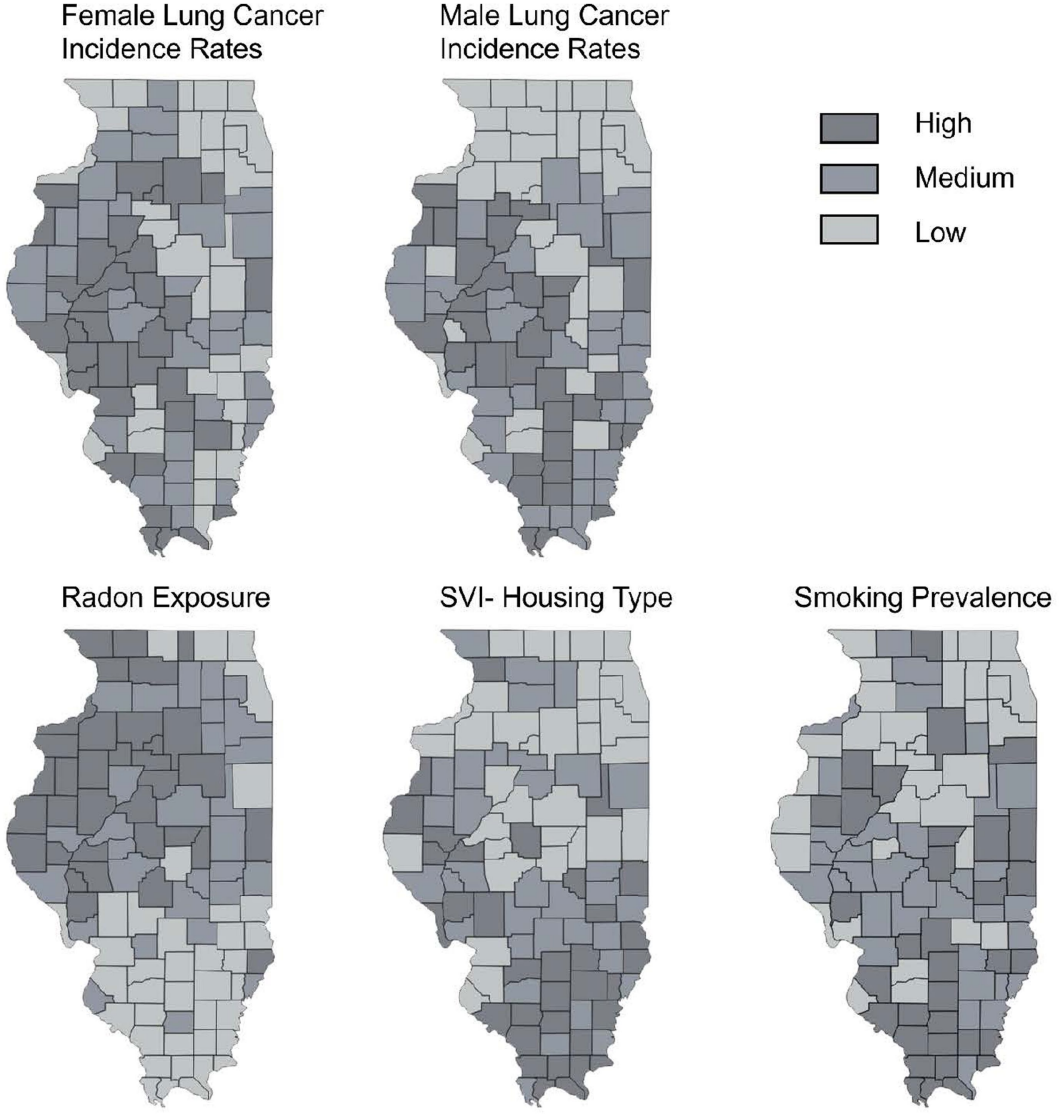
Lung cancer risk factors and incidence rates in Illinois for males and females using univariate choropleth mapping spatial analyses. Counties were classified into three equal groups (tertiles) based on the distribution of data across Illinois, including Low: Bottom 33.33% of counties (lowest risk/ prevalence), Medium: Middle 33.33% of counties (middle risk/prevalence), and High: Top 33% of counties (highest risk/prevalence). This relative classification highlights geographic disparities for the three risk factors (radon, smoking, Social Vulnerability Index, SVI-Housing Type) for male and female lung cancer incidence rates within the state of Illinois.

To visualize spatial relationships, we also conducted geospatial analyses in ArcGIS Pro 2.9 and Tableau Public software to facilitate interpretation and practical application, alongside our constructed regression models. Tableau Public is a free visualization and data analysis tool that leverages its intuitive design and powerful capabilities for map creation and visualization without requiring code. Univariate and Bivariate Choropleth Mapping Analyses were performed to simultaneously examine lung cancer incidence and each of the three exposures (radon, smoking, and housing & transportation vulnerability) as social and environmental risk factors. Each targeted variable was divided into tertiles (low, medium, high), resulting in nine possible combinations. County shapefiles were obtained from the U.S. Census Bureau’s TIGER/Line database and merged with relevant epidemiologic data for this study. As shown in Figure 1, counties identified as ‘High-High’ (HH), defined as those in the highest tertile for both lung cancer incidence and a specific environmental exposure, were designated as the highest priority regions for intervention. Further analytical strategies to identify the second-highest priority areas for funding and resource allocation considered counties classified as ‘Medium-High’ (MH), which have middle tertile radon exposure and highest tertile cancer incidence, as well as ‘High-Medium’ (HM), characterized by highest tertile radon exposure and middle tertile cancer incidence.”

## 3 Results

Age-adjusted lung cancer incidence rates averaged 85.20 per 100,000 population (SD = 21.63) for males and 66.09 per 100,000 population (SD = 13.41) for females across Illinois counties. Adult smoking prevalence averaged 15.66% (SD = 1.52%), while mean residential radon levels were 4.69 picocuries per liter (pCi/L) (SD = 2.06). The housing-related Social Vulnerability Index (SVI) has a mean score of 1.09 (SD = 0.29).

We conducted separate multivariate regression analyses for male and female lung cancer incidence rates and concluded the final model below:


$$\begin{array}{l} \text{Lung Cancer Incidence}=\beta_{0}+\beta_{1}\times (\text{Smoking Prevalence})+\\ \beta_{2}\times (\text{Housing Vulnerability})+\\ \beta_{3}\times (\text{Radon Exposure})+\varepsilon , \end{array}$$

where $\beta_{0}$ represents the intercept, $\beta_{1-3}$ represent the regression coefficients for each environmental factor, and *ε* represents the error term.

To validate the assumption of independence, we tested the regression residuals for spatial autocorrelation using Moran’s I statistic for males and females. The analysis for males yielded a Moran’s I of 0.038 (*p* = 0.235), indicating that the residuals were spatially random and the independence assumption was met. The regression model (Table 2, second part) for male lung cancer incidence demonstrated that for each percentage point increase in smoking prevalence, the lung cancer rate increased by approximately 7.2 cases per 100,000 population, holding other variables constant. For the Social Vulnerability Index related to housing, each unit increase corresponded to an increase in male lung cancer rates. For female lung cancer incidence, each percentage point increase in smoking prevalence was associated with an increase of approximately 5.1 cases per 100,000 population. Additionally, each unit increase in radon exposure (pCi/L) was associated with an increase in female lung cancer rates.

**Table 2:** Regression coefficients of environmental factors on Illinois lung cancer incidence rates.

Variable	***β***	SE	*B*	*t*	*p*	VIF
Female						
Constant	−30.066	12.915		−2.328	0.022	
Radon exposure	1.266	0.565	0.195	2.242	0.027	1.186
Smoking prevalence	5.215	0.771	0.593	6.768	<0.001	1.205
Social vulnerability index – housing type	6.883	4.002	0.150	1.720	0.089	1.193
*R^2^*	0.377					
Δ*R^2^*	0.358					
*F*	19.735					
AIC	779.908					
BIC	793.032					
Male						
Constant	−59.277	20.891		−2.837	0.006	
Radon exposure	1.473	0.914	0.140	1.612	0.110	1.186
Smoking prevalence	7.570	1.246	0.533	6.074	<0.001	1.205
Social vulnerability index – housing type	17.333	6.473	0.234	2.678	0.009	1.193
*R^2^*	0.373					
Δ*R^2^*	0.354					
*F*	19.425					
AIC	876.008					
BIC	886.508					

*N* = 102. Social Vulnerability Index – Housing Type (% of mobile homes and % group-quarters population).

Multivariate regression analyses revealed significant associations between environmental factors and lung cancer incidence rates, with sex differences across Illinois counties (Table 2). Both models explained approximately 37% of the variance in lung cancer rates (*p* < 0.001). Smoking prevalence emerged as the strongest predictor for both sexes. Radon exposure was a significant predictor for females ( β = 0.195, *p* = 0.027), but not for males. In contrast, the SVI related to housing type was significant for males ( β = 0.234, *p* = 0.009), but not for females.

Given the significant autocorrelation result for females (Moran’s I = 0.209, *p* = 0.002), we need to examine and evaluate the covariates in the female regression model more carefully. The Spatial Error Model (SEM) was also fitted for females, and the results are reported in Table 3. The SEM provided a superior fit to the OLS model, as indicated by lower AIC and BIC values and a significant spatial error parameter (*λ* = 0.389, *p* = 0.002), demonstrating that female lung cancer incidence rates exhibit spatial clustering across Illinois counties. After accounting for spatial dependence, smoking prevalence remained a strong and statistically significant predictor; radon exposure showed a marginal association, while the housing-related Social Vulnerability Index did not reach statistical significance. In brief, radon exposure showed a significant positive association with female lung cancer rates in the OLS model, but became marginally significant in the Spatial Error Model, with the magnitude of the effect remaining nearly unchanged. This suggests that radon may contribute to female lung cancer incidence, but part of its association is shared with broader spatially structured factors across counties.

**Table 3:** Comparison of ordinary least squares (OLS) and spatial error model (SEM) Results for female lung cancer incidence rates.

Predictor	OLS Model		Spatial Error Model (SEM)	
	B (SE)	*p*	B (SE)	*P*
Constant	−30.066 (12.915)	0.022	−24.724 (12.808)	0.054
Radon exposure	1.266	0.027	1.188	0.057
Smoking prevalence	5.215	<0.001	4.881	<0.001
Social vulnerability index – housing type	6.883	0.089	7.099	0.091
Spatial parameters				
Lambda (λ)^a^			0.389	0.002
Model Fit				
R^2^ (Adjusted)	0.377		0.376^b^	
Log Likelihood	−384.954		−380.501	
AIC	779.908		769.002	
BIC	793.032		779.502	

*N* = 102. Dependent variable: Lung Cancer Incidence Rate (per 100,000). Social Vulnerability Index – Housing Type (e.g., multi-unit housing, crowding, mobile home, no vehicle available). ^a^(λ) represents the spatial autoregressive coefficient for the error term indicating significant spatial clustering of unexplained variance (*p* = 0.002). ^b^Pseudo R-squared reported for SEM. Lower AIC and BIC values in the SEM indicate superior model fit compared to OLS.

Furthermore, given the potential impact of air pollution on cancer outcomes, we evaluated the inclusion of air pollution metrics, specifically Ozone and PM2.5, in our regression models to enhance predictive accuracy. However, these variables did not contribute statistically significant improvements to model robustness in the context of our regional spatial analysis in Illinois. For the male population, the full regression model with five predictors achieved higher AIC (879.762 vs. 876.008) and higher BIC (898.137 vs. 886.508) values than the reduced model (with three predictors), indicating poorer overall model fit. In addition, the female’s full model showed higher AIC (782.055 vs. 779.908) and higher BIC (800.430 vs.793.032) than the reduced model. Consequently, they were excluded from the final model specifications, reflecting the rigorous model selection process in which only predictors with demonstrable significance are retained.

**Figure 2:**
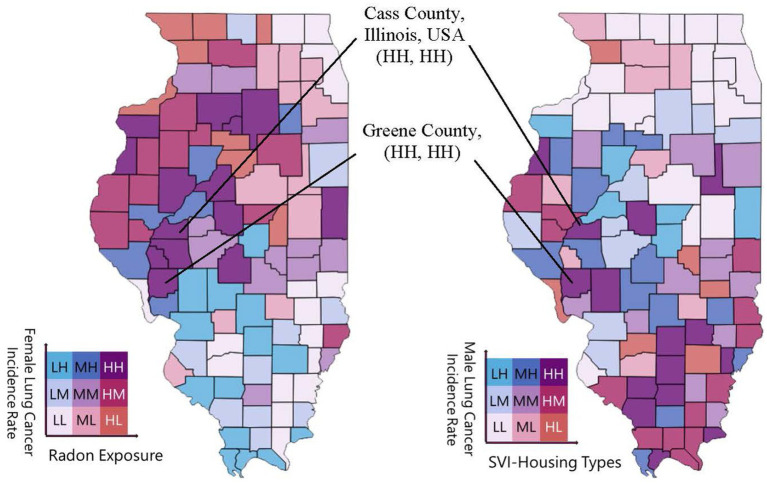
Gender-stratified bivariate analyses of lung cancer incidence and environmental risk in Illinois (Demonstration of Counties with Highest Risks). These two side-by-side geospatial mapping analytical results depict joint tertile classifications of lung cancer incidence and one of three levels of lung cancer risk factors in present study: indoor radon exposure, smoking prevalence, or the housing-related Social Vulnerability Index (SVI). For this SVI indicator, each county receives a percentile ranking for this theme. Percentile ranks range from 0% (least vulnerable) to 100% (most vulnerable). Counties are categorized into nine combinations using tertile cutoffs (L = Low, M = Medium, H = High), with “HH” zones indicating co-occurrence of high cancer burden and high exposure to lung cancer environmental risk factors.

**Figure 3:**
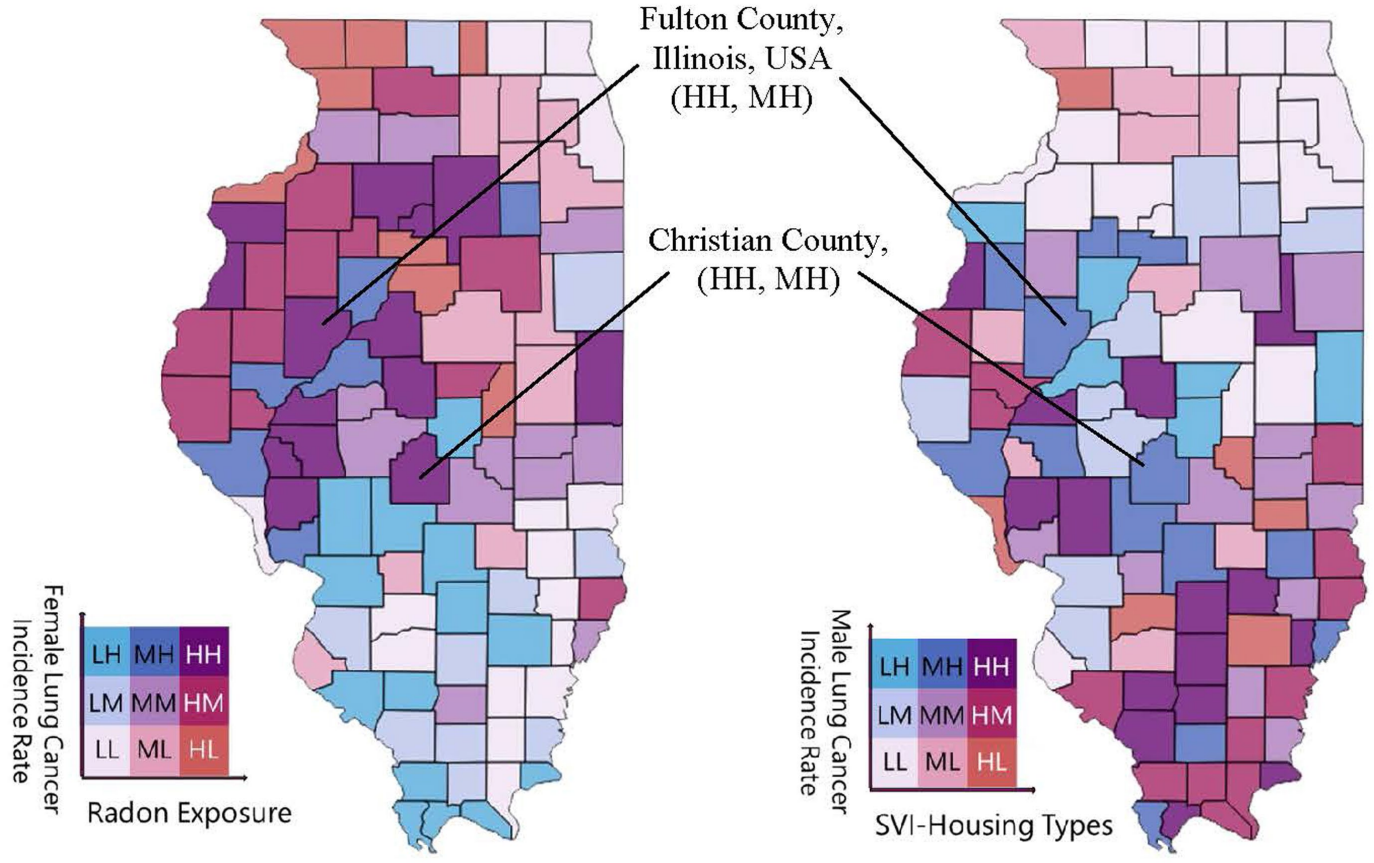
Gender-stratified bivariate analyses of lung cancer incidence and environmental risk in Illinois. Two side-by-side gender-stratified bivariate choropleth maps show the joint classification (tertiles: Low, Medium, High) of lung cancer incidence and one of three risk factors: indoor radon, smoking prevalence, or the housing-related Social Vulnerability Index (SVI). Counties are grouped into nine color classes of cancer risk evaluation units. The higher risk zones (‘HH’, ‘MH’), exemplified by counties such as Fulton and Christian County, are specifically highlighted on the maps as priority areas for intervention.

The univariate choropleth mapping analyses (Figure 1) provided fundamental information for each lung cancer risk factor separately before combining them into bivariate choropleth maps. Bivariate geospatial analyses (i.e., Bivariate Choropleth Mapping Analyses) in Figures 2, 3 revealed distinct patterns in lung cancer incidence rates by sex and their associations with radon exposure and SVI housing types. For females, the northwestern region of Illinois (also see Table 4) emerged as a primary high-priority zone (shown as several ‘HH’ classifications), with counties exhibiting both high lung cancer incidence rates and elevated radon exposure levels (Figure 2; Table 5). For male lung cancer incidence and its relationship with SVI-housing types, patterns were more dispersed, with central and eastern Illinois counties (also see Table 6) showing high male lung cancer rates, coinciding with significant housing vulnerability concerns. The southern region displayed different patterns between the two maps, generally showing lower female lung cancer rates and radon exposure, but mixed levels of male lung cancer rates and housing vulnerability.

**Table 4:** Bivariate classification of Illinois counties based on residential radon levels and female lung cancer incidence rates.

Spatial cluster group (Radon with female lung cancer ranking)	Illinois counties
HH	Bureau, Cass, Christian, Fulton, Greene, Henderson, La Salle, Logan, Mercer, Morgan, Putnam, Scott, Tazewell, Vermilion
HM	Adams, Brown, DeWitt, Hancock, Henry, Knox, Lawrence, Livingston, McDonough, Ogle, Stark, Warren
HL	Boone, Carroll, Jo Daviess, Marshall, Piatt, Rock Island, Stephenson, Woodford
MH	Fayette, Grundy, Jersey, Macon, Mason, Peoria, Pike, Schuyler
MM	Coles, Douglas, Edgar, Franklin, Iroquois, Kankakee, Lee, Menard, Moultrie, Sangamon, Shelby, Wabash, Whiteside, Winnebago
ML	Bond, Champaign, Clinton, DeKalb, DuPage, Effingham, Ford, Kane, Kendall, McLean, Monroe, Will
LH	Alexander, Hardin, Macoupin, Madison, Marion, Massac, Montgomery, Perry, Pulaski, Randolph, Union, Wayne
LM	Clay, Crawford, Gallatin, Jackson, Jefferson, Johnson, St Clair, Williamson
LL	Calhoun, Clark, Cook, Cumberland, Edwards, Hamilton, Jasper, Lake, McHenry, Pope, Richland, Saline, Washington, White

**Table 5:** Intersection of highest-risk or higher-risk areas: Illinois counties classified as High-High (HH) or High-Medium (HM) in both radon exposure for females and SVI-housing type for males bivariate choropleth mapping analyses.

County	Radon exposure & female lung cancer incidence rate	SVI-housing type & male lung cancer incidence rate
Cass	HH	HH (see Figure 2)
Greene	HH	HH (see Figure 2)
Christian	HH	MH (see Figure 3)
Fulton	HH	MH (see Figure 3)

HH, High exposure/High incidence; HM, High exposure/Medium incidence; HL, High exposure/Low incidence.

**Table 6:** Bivariate classification of Illinois counties based on social vulnerability index (SVI) housing type and male lung cancer incidence rates.

Spatial cluster group (Social vulnerability index – housing type with male lung cancer ranking)	Illinois counties
HH	DeWitt, Franklin, Fulton, Jackson, Jefferson, Macon, Macoupin, Marion, Peoria, Vermilion, Williamson
HM	Adams, Coles, Kankakee, Knox, La Salle, Madison, Randolph, Saline, Sangamon, St Clair, Tazewell
HL	Champaign, Cook, DuPage, Kane, Lake, McHenry, McLean, Ogle, Rock Island, Whiteside, Will, Winnebago
MH	Cass, Christian, Clay, Fayette, Ford, Greene, Logan, Mason, Montgomery, Morgan, Perry
MM	Crawford, Edgar, Grundy, Hancock, Iroquois, Jersey, Lawrence, Livingston, Shelby, Union, White
ML	Boone, Bureau, Carroll, Clinton, Effingham, Henry, Jo Daviess, Lee, McDonough, Stephenson, Wayne, Woodford
LH	Alexander, Edwards, Hardin, Henderson, Jasper, Marshall, Mercer, Pike, Pulaski, Stark, Wabash, Warren
LM	Bond, Brown, Clark, Douglas, Gallatin, Hamilton, Johnson, Massac, Menard, Pope, Richland, Schuyler
LL	Calhoun, Cumberland, DeKalb, Kendall, Monroe, Moultrie, Piatt, Putnam, Scott, Washington

As we explained earlier in the Data and Methods section, our bivariate choropleth mapping approach classified counties into tertiles (Low, Medium, High) for both cancer incidence rates and environmental exposures, creating a 3 × 3 matrix of possible combinations. This visualization revealed that 15 counties (14.7%) fell into the ‘HH’ category for female lung cancer and radon exposure, primarily clustered in the state’s northwestern region. For male lung cancer and housing vulnerability, 12 counties (11.8%) were classified as ‘HH,’ with a more dispersed pattern across central and eastern Illinois. The differential spatial patterns observed between male and female lung cancer rates due to environmental exposures suggest potential sex-specific vulnerabilities to environmental risk factors.

**Figure 4:**
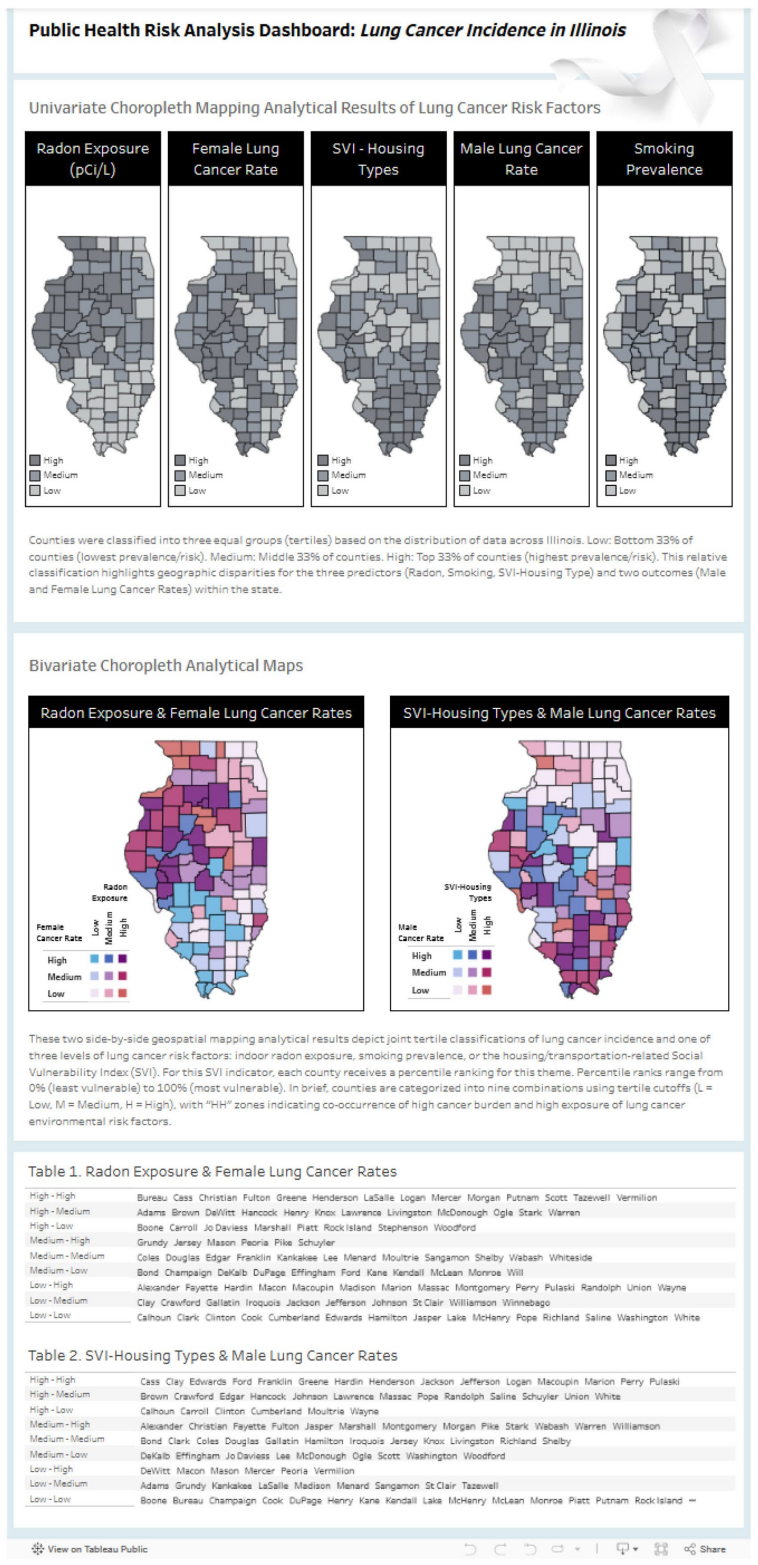
An interactive dashboard of gender-stratified bivariate analyses of lung cancer incidence and risk factors in Illinois. The interactive dashboard visualizes the simultaneous distribution of lung cancer incidence rates (low, medium, high tertiles) and three environmental and social risk factors: radon exposure, smoking prevalence, and housing & transportation vulnerability. County-level data from the U. S. Census Bureau TIGER/Line database were classified into tertiles to create nine possible combinations of risk exposure and disease burden. The color-coded legend represents the following priority-ranked county classifications: High-High (HH, darkest hue), indicating highest tertile values for both lung cancer incidence and a specific environmental exposure, designating these counties as the highest priority regions for targeted public health interventions and resource allocation; Medium-High (MH), characterized by middle tertile environmental exposure and highest tertile cancer incidence; and High-Medium (HM), characterized by highest tertile environmental exposure and middle tertile cancer incidence, representing secondary priority areas for intervention planning. The dashboard design allows public health practitioners, policymakers, and resource allocation decision-makers to rapidly identify geographic “hotspot” regions where lung cancer risk is compounded by environmental and social vulnerability factors, thereby supporting evidence- based prioritization of prevention, screening, and treatment interventions.

Finally, we developed an interactive dashboard (Figure 4) to help readers maximize the use of integrated visual data alongside our findings for public health planning. The link/URL of the dashboard (https://go.illinois.edu/lung [it will be inserted during the copyediting stage, which can avoid revealing the authors’ information and identity]) provides public access and open-source usability. The interactive dashboard visualizes the simultaneous distribution of lung cancer incidence rates (low, medium, high tertiles) and three environmental and social risk factors: radon exposure, smoking prevalence, and housing & transportation vulnerability. The dashboard design allows public health practitioners, policymakers, and resource allocation decision-makers to rapidly identify geographic “hotspot” regions where lung cancer risk is compounded by environmental and social vulnerability factors, thereby supporting evidence-based prioritization of prevention, screening, and treatment interventions.

## 4 Discussion

### 4.1 Principal findings

This study provides new insights into how environmental exposures and housing-related social vulnerability jointly contribute to sex-specific patterns in lung cancer incidence across Illinois counties. By combining geospatial mapping with multivariate regression, we identified distinct risk profiles for males and females, highlighting how social determinants of health (SDOH) and lung cancer risk factors shape environmental exposure and cancer disparities in specific ways through the innovative integration of model building and a unique spatial analysis approach.

Our findings confirm and extend previous research showing that smoking remains the most potent predictor of lung cancer incidence for both sexes (3). However, this study moves beyond prior work by demonstrating that radon exposure significantly contributes to female lung cancer rates, independent of smoking prevalence. In contrast, housing-related vulnerability is more predictive of male lung cancer risk. These findings reinforce the importance of adopting sex-specific lenses when evaluating environmental cancer risks (16, 21, 22, 32).

Among females, the significant association between radon exposure and lung cancer incidence aligns with earlier studies suggesting that women may be more biologically or behaviorally susceptible to indoor environmental carcinogens (16). The spatial clustering of high female lung cancer rates and high radon exposure in northwestern Illinois underscores the need for targeted interventions, particularly in residential areas with historically elevated radon levels and limited mitigation uptake. As previous work shows, women—especially those in rural or economically disadvantaged communities—may spend more time indoors and face structural barriers to radon testing and remediation (9, 10, 33). These findings support the expansion of public health initiatives that prioritize accessible radon testing, remediation subsidies, and education campaigns targeting high-risk populations, particularly women.

In contrast, for males, housing-related social vulnerability was a stronger predictor than radon exposure. This suggests that structural factors—such as substandard housing, residential crowding, or lack of infrastructure—may play a larger role in driving lung cancer risk for men. These patterns may reflect occupational exposures, housing-linked environmental hazards, or cumulative disadvantage among socially vulnerable male populations (19, 22, 26, 28, 29). Geospatial analysis identified counties in central and eastern Illinois as high-priority areas for male-specific interventions (34). Public health responses in these areas should consider the role of housing code enforcement, community development, and workplace exposure mitigation.

Biological mechanisms, such as the interaction between estrogen receptors and carcinogen metabolism, or the higher prevalence of specific genetic mutations, such as epidermal growth factor receptor (EGFR), in women, may underpin the distinct susceptibility of women to environmental hazards such as radon (7, 15). Furthermore, gender-specific behavioral patterns suggest that women may have historically spent more time indoors, thereby increasing their exposure to residential radon compared to men. Conversely, the strong association between male lung cancer and housing vulnerability likely reflects gendered occupational roles and structural exposures, where men are disproportionately subjected to cumulative environmental hazards found in economically disadvantaged settings.

Together, these findings illustrate the interplay between environmental risks and structural inequities, reinforcing the need for precision public health approaches that integrate sex, place, and social context. By leveraging tools like the Social Vulnerability Index (SVI), stakeholders can more effectively allocate resources, tailor prevention strategies, and reduce disparities in cancer burden (18, 25, 27, 35, 36). These results also advance the environmental justice agenda by identifying populations and places disproportionately affected by modifiable environmental risks (23, 24, 37).

The sex-specific associations found in this study suggest that universal approaches to cancer prevention may overlook critical nuances. For females, addressing environmental exposures such as radon may have a greater impact, whereas for males, tackling the structural drivers of vulnerability—particularly those linked to housing—may yield more meaningful reductions in risk. For both groups, continued investment in smoking cessation programs remains essential (38, 39). Additionally, integrating indoor environmental monitoring and housing interventions into broader cancer control strategies may enhance their effectiveness and equity.

Geospatial analysis can play a crucial role in identifying high-burden counties, providing visual evidence to support data-driven decision-making. By classifying counties into ‘high-high’ (HH) zones based on both cancer rates and environmental risk levels, we identified geographic areas (e.g., counties with poorer social determinants of health indicators) and regions that are most in need of immediate action (Figure 2; Table 5). For example, counties in northwestern Illinois emerged as radon-priority zones for females, while housing-vulnerable counties in central and eastern Illinois were highlighted for male-focused efforts. These regionally tailored strategies can help overcome resource fragmentation and promote coordinated local responses.

To further refine priority setting across sex-specific subgroups, an integrated approach can be undertaken by combining the results of bivariate choropleth mapping for both females and males. This joint spatial analysis enables comprehensive identification of counties that warrant targeted interventions for both populations. For instance, Fulton and Christian Counties exhibited a ‘High-High’ (HH) classification for lung cancer incidence among women and a ‘High-Medium’ (HM) classification among men (Figure 3; Table 5). These findings underscore Fulton and Christian Counties as another priority areas for addressing elevated lung cancer rates and related social vulnerabilities, highlighting opportunities for coordinated, evidence-based resource allocation.

In summary, our study highlights the importance of segmenting different populations based on specific geographic and demographic variables using data modeling techniques to reduce the disease burden. Cancer reduction strategies should consider effective risk communication, which may require multiple exposures to a message (40), and radon remediation in new housing, as mitigation in existing homes is not cost-effective as a public policy (41). Long-term inequities in housing in vulnerable areas should be addressed in state and federal policy.

### 4.2 Study limitations

This study used validated, publicly available data sources and applied robust geospatial and regression methods. Nonetheless, several limitations should be acknowledged. First, as an ecological study, the analysis cannot infer individual-level causality. Second, some variables—such as radon exposure and smoking—were based on county-level aggregates, which may mask variation within counties. Third, we utilized historical radon data (2003–2019) to reflect long-term exposure; however, measurement timing may vary across counties.

Moreover, the SVI housing indicator captures structural vulnerability broadly, but not all individual components of housing quality. Finally, we acknowledge the temporal mismatch between the long latency period of lung cancer, typically spanning one to three decades, and the averaged nature of our exposure data. Using lung cancer incidence rates from 2017 to 2021 alongside radon data aggregated from 2003 to 2019 assumes a degree of spatial stability in risk factors that may not fully capture individual long-term exposure histories. However, previous cohort studies indicate that the excess relative risk from radon exposure peaks approximately 5 to 14 years after exposure and then declines over subsequent decades (42, 43). Consequently, our radon dataset from 2003–2019 might cover the critical peak-risk etiological window for cases diagnosed in 2017–2021.

Another aspect of limitation also involves the temporal alignment of the exposure data. We utilized contemporary Radon exposure measurements to explain current lung cancer rates. However, lung cancer typically consists of a latency period of 20 to 30 years. While we assume geological radon levels remain relatively stable over time, we acknowledge this as a limitation of the present study. Contemporary levels serve as a proxy for the historical exposure that would have initiated the oncogenic process.

Furthermore, this ecological approach might miss the effects of exposures incurred 30 or more years prior. This limitation is particularly relevant for older populations, where the risk decline is steeper, or for newer housing stock, where radon concentrations may be trending upward (42, 44). Therefore, our findings should be interpreted as associations with the cumulative environmental and social context of these counties rather than a precise reconstruction of historical dose–response relationships.

### 4.3 Future research directions

Despite these limitations, this study offers a valuable framework for investigating lung cancer disparities by integrating spatial and statistical methods. Future research should investigate longitudinal patterns of environmental exposure, incorporate more detailed data (e.g., ZIP code or census tract), and examine the interactions between housing policy, environmental monitoring, and health outcomes. Expanding this approach to other states or urban–rural contexts could uncover additional geographic disparities and guide localized cancer prevention strategies. Importantly, future work should also consider racial and ethnic inequalities alongside sex, given their intersectional effects on vulnerability and exposure.

## 5 Conclusion

This study identified sex-specific associations between environmental and social risk factors within the lens of social determinants of health (SDOH)—namely, radon exposure, smoking prevalence, and housing-related vulnerability—and lung cancer incidence across Illinois counties. These findings highlight the need for regionally and demographically tailored interventions that promote environmental health equity. By integrating sex, place, and SDOH into analytic frameworks and public health planning, we can better target prevention resources, reduce disparities, and advance environmental justice in cancer control.

## Data Availability

Publicly available datasets were analyzed in this study. This data can be found here: https://ilema.maps.arcgis.com/apps/dashboards/93e4f4824a4046b48d1f35b7226069a8.
