# Supplementary material for: Gender disparities in lung cancer and social determinants of environmental risk: a geospatial analysis across Illinois counties
Source: Front Public Health. 2026 Jan 23;13:1676853. doi: 10.3389/fpubh.2025.1676853 (PMC12876219; doi:10.3389/fpubh.2025.1676853)
Supplement: Supplementary file 1 [file Table_1.DOCX]

**Supplementary S1: Map of EPA Radon Zones in Illinois**


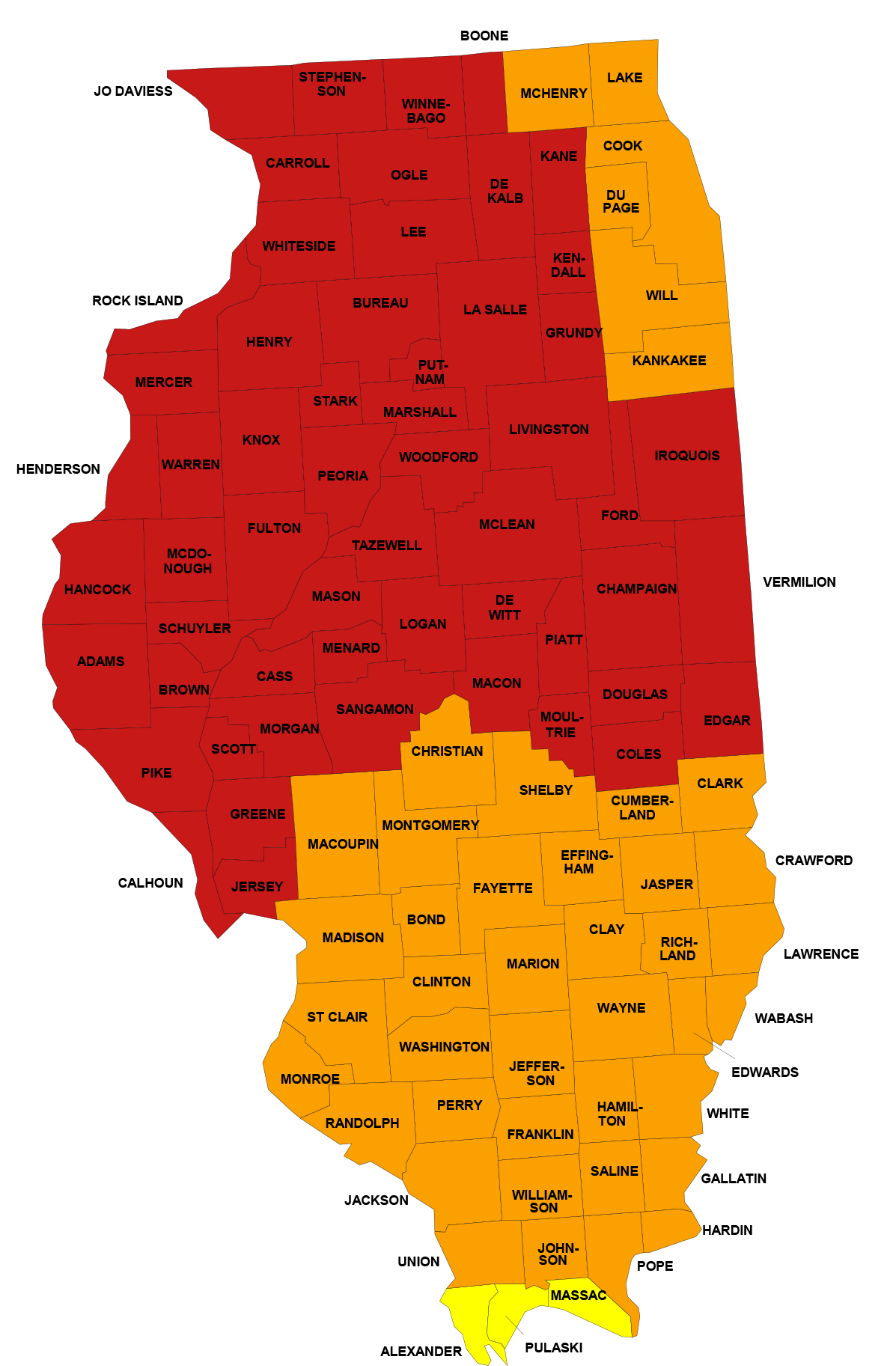


**Supplementary S1.** Map of EPA Radon Zones in Illinois

What do the colors mean?

| Color | Zone | Description |
| --- | --- | --- |
|  | Zone 1  (red zones) | Highest potential: Counties have a predicted average indoor screening level > (Greater) than 4 pCi/L (picocuries per liter) |
|  | Zone 2  (orange zones) | Moderate potential: Counties have a predicted average screening level ≥ (Greater than and equal to) 2 pCi/L (75 Bq/m3) and ≤ (less than and equal to) 4 pCi/L |
|  | Zone 3  (yellow zones) | Low potential: Counties have a predicted average indoor screening level < (Less than) 2 pCi/L |

Source: U.S. Environmental Protection Agency (https://www.epa.gov/sites/default/files/2015-03/documents/illinois.pdf)
