# Supplementary material for: Gender disparities in lung cancer and social determinants of environmental risk: a geospatial analysis across Illinois counties
Source: Front Public Health. 2026 Jan 23;13:1676853. doi: 10.3389/fpubh.2025.1676853 (PMC12876219; doi:10.3389/fpubh.2025.1676853)
Supplement: Supplementary file 2 [file Table_2.DOCX]

**Supplementary S2: Residual Diagnostics of Lung Cancer Prediction Regression Model**

**
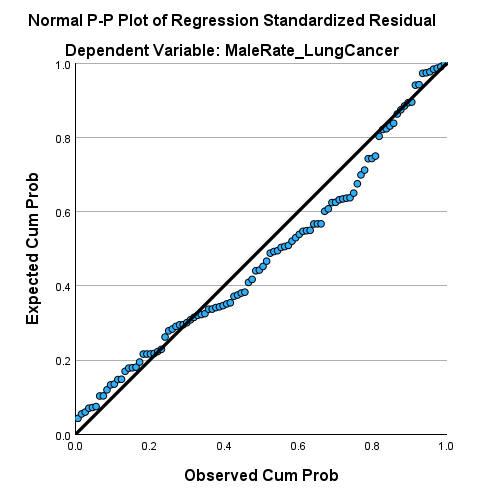
**

**Supplementary S2 Residual Diagnostics #1.** Q-Q Plot (Normal P-P Plot) of the Lung Cancer Prediction Model for Males in Illinois

*Note.* The normality of residuals was examined using a Quantile-Quantile (Q-Q) plot. The residuals followed the 45-degree reference line closely, suggesting an approximate normal distribution. While minor deviations were observed, the regression results are still acceptable (n>30). Thus, the inference (p-values and CIs) remains valid for the male’s OLS model.


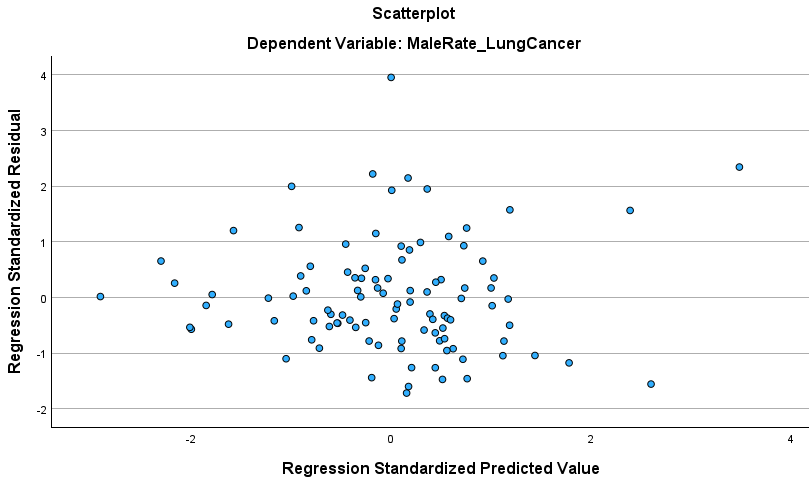


**Supplementary S2 Residual Diagnostics #2.** Homoscedasticity Examination of the Lung Cancer Prediction Model for Males in Illinois

*Note.* This assumption was evaluated using visual diagnostics. This scatterplot (i.e., Standardized Residual vs. Standardized Predicted Value Plot) shows a random scatter of points with no distinct funnel or fan shape. It also shows a relatively horizontal trend line with data points spread uniformly, indicating consistent variance across prediction levels. The OLS estimator remains efficient, and the confidence intervals are reliable without the need for robust standard error (SE) corrections. In conclusion, the assumption of homoscedasticity is met.
